# Supplementary material for: Designing diverse and high-performance proteins with a large language model in the loop
Source: PLoS Comput Biol. 2025 Jun 5;21(6):e1013119. doi: 10.1371/journal.pcbi.1013119 (PMC12169546; doi:10.1371/journal.pcbi.1013119)
Supplement: S1 Text — (PDF) [file pcbi.1013119.s001.pdf]

S1 Text. Supporting Information

Supporting Information Legend

This file contains supplementary figures, tables, and methodological details referenced in the main text.

S1 Text. Appendix A: Seq2Fitness performance on specific datasets and data splits.

Table A in S1 Text. Performance of Seq2Fitness and alternative models with random split

Model	AAV	GB1	AMY_BACSU	NucB	Average
Zero-shot	0.05	0.08	0.61	0.33	0.27
Linear one-hot	0.80	0.79	0.65	0.71	0.74
Linear ESM	0.86	0.86	0.65	0.80	0.79
Aug. linear	0.81	0.79	0.65	0.77	0.75
CNN one-hot	0.91	0.92	0.72	0.92	0.87
CNN AAindex	0.91	0.92	0.72	0.92	0.87
CNN ESM	**0.92**	0.92	0.38	0.91	0.78
Seq2Fitness	0.91	**0.93**	**0.75**	**0.92**	**0.88**

Table B in S1 Text. Performance of Seq2Fitness and alternative models with two-vs-rest split

Model	AAV	GB1	AMY_BACSU	NucB	Average
Zero-shot	0.15	0.11	0.54	0.45	0.31
Linear one-hot	0.66	0.57	0.57	0.37	0.54
Linear ESM	0.72	0.46	0.44	0.55	0.54
Aug. linear	0.66	0.58	0.58	0.46	0.57
CNN one-hot	0.69	0.53	0.23	0.49	0.48
CNN AAindex	0.51	0.32	0.07	**0.66**	0.39
CNN ESM	0.45	0.52	0.33	0.52	0.46
Seq2Fitness	**0.77**	**0.62**	**0.61**	0.62	**0.66**

Table C in S1 Text. Performance of Seq2Fitness and alternative models with mutational split

Model	AAV	GB1	AMY_BACSU	NucB	Average
Zero-shot	0.19	-0.39	0.62	0.10	0.13
Linear one-hot	0.61	0.57	0.52	0.42	0.53
Linear ESM	0.78	0.05	0.06	0.53	0.35
Aug. linear	0.61	0.21	0.62	0.46	0.47
CNN one-hot	0.74	0.48	0.34	0.23	0.45
CNN AAindex	0.84	0.58	0.28	**0.68**	0.59
CNN ESM	0.67	0.45	0.17	0.32	0.40
Seq2Fitness	**0.82**	**0.69**	**0.71**	0.64	**0.72**

Table D in S1 Text. Performance of Seq2Fitness and alternative models with positional split. Positional splits with GB1 did not yield sufficient number of sequences for evaluation, and was consequently dropped from the analyses.

Model	AAV	GB1	AMY_BACSU	NucB	Average
Zero-shot	0.17	NA	**0.66**	0.20	0.34
Linear one-hot	0.01	NA	0.02	-0.30	-0.09
Linear ESM	0.26	NA	0.08	0.45	0.26
Aug. linear	0.11	NA	0.52	0.30	0.31
CNN one-hot	0.16	NA	0.35	-0.28	0.08
CNN AAindex	0.45	NA	0.07	0.17	0.23
CNN ESM	0.10	NA	0.15	-0.39	-0.05
Seq2Fitness	**0.53**	NA	0.64	**0.48**	**0.55**

Table E in S1 Text. Ablation of Seq2Fitness with random split

Model	AAV	GB1	AMY_BACSU	NucB	Average
Seq2Fitness	**0.91**	**0.93**	**0.75**	**0.92**	**0.88**
w/ raw embeddings	0.90	0.87	0.57	0.92	0.82
w/o embeddings	0.72	0.55	0.57	0.80	0.66
w/o zero-shot scores	0.91	0.92	0.73	0.92	0.87
w/o normalized scores	0.91	0.92	0.74	0.92	0.88
w/o log-probabilities	0.91	0.93	0.75	0.92	0.88

Table F in S1 Text. Ablation of Seq2Fitness with two-vs-rest split

Model	AAV	GB1	AMY_BACSU	NucB	Average
Seq2Fitness	0.77	**0.62**	**0.61**	0.62	**0.66**
w/ raw embeddings	0.75	0.42	0.43	0.55	0.54
w/o embeddings	0.55	0.22	0.43	0.08	0.32
w/o zero-shot scores	0.73	0.61	0.58	0.64	0.64
w/o normalized scores	0.78	0.62	0.57	**0.66**	0.66
w/o log-probabilities	**0.78**	0.60	0.52	0.60	0.62

Table G in S1 Text. Ablation of Seq2Fitness with mutational split

Model	AAV	GB1	AMY_BACSU	NucB	Average
Seq2Fitness	**0.82**	**0.69**	**0.71**	0.64	**0.72**
w/ raw embeddings	0.79	0.51	0.65	**0.73**	0.67
w/o embeddings	0.66	-0.13	0.65	0.66	0.46
w/o zero-shot scores	0.80	0.67	0.70	0.62	0.70
w/o normalized scores	0.82	0.69	0.71	0.63	0.71
w/o log-probabilities	0.82	0.68	0.71	0.59	0.70

Table H in S1 Text. Ablation of Seq2Fitness with positional split

Model	AAV	GB1	AMY_BACSU	NucB	Average
Seq2Fitness	**0.53**	N/A	0.64	**0.48**	**0.55**
w/ raw embeddings	0.51	N/A	0.73	0.12	0.45
w/o embeddings	0.42	N/A	0.65	0.44	0.51
w/o zero-shot scores	0.51	N/A	0.70	0.27	0.49
w/o normalized scores	0.49	N/A	**0.75**	0.20	0.48
w/o log-probabilities	0.49	N/A	0.75	0.33	0.52

S1 Text. Appendix B: Detailed results on BADASS amylase and NucB tasks, and comparison to alternate approaches.

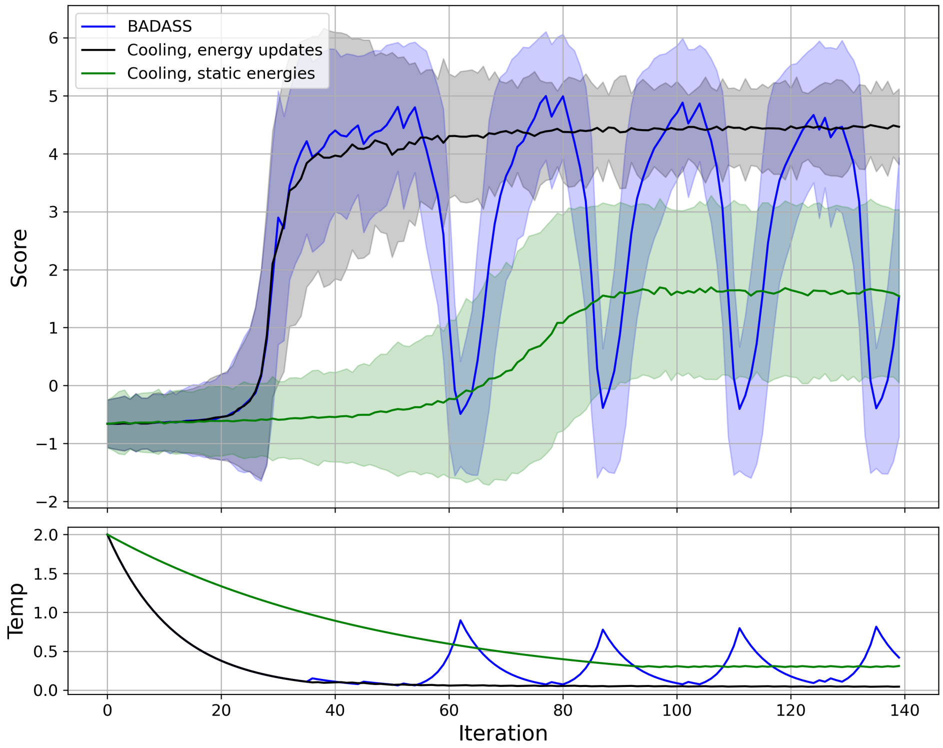

Fig A in S1 Text. Comparison of BADASS with an approach that simply cools while updating the mutation energies like BADASS does (black), and with an approach that also simply cools but does not update the mutation energies (green) for the Seq2Fitness alpha-amylase task to explore sequences with 6 mutations. Mutation energies for all approaches are initialized to the single mutant scores, with a batch size of 1,000 sequences per iteration. The score envelopes denote the mean $\pm 1.96\sigma$. The oscillations of BADASS maintain a high variance even when the average score is high, leading to a score envelope that is clearly higher than without the temperature-driven oscillations. Removing the mutation energy updates results in convergence to mediocre scores. The non-BADASS approaches here use minor re-heating when the temperature gets so low that the sampler fails to sample at least half the desired sequence batch size.

Table I in S1 Text. Fuller BADASS results on alpha amylase tasks. The entropies are shown as the percentage relative to the maximum entropy of a uniform distribution.

		**Gamma = 0**						**Gamma = 1**					
Fitness	Metric	**k = 2-6**	**2**	**3**	**4**	**5**	**6**	**2-6**	**2**	**3**	**4**	**5**	**6**
**ESM2**	% better than WT	**100**	**100**	**100**	**100**	**100**	**100**	**100**	**100**	**100**	**100**	**100**	**100**
	Best score	55.36	20.19	30.21	39.00	47.12	53.91	**55.91**	20.19	30.21	39.00	47.85	54.12
	100th best score	49.49	16.52	26.45	36.29	43.50	49.76	**52.24**	16.52	26.54	36.37	45.62	52.09
	1000th best score	41.84	14.32	24.35	33.68	40.03	46.68	44.10	14.33	24.58	33.90	42.20	**50.28**
	Unique mutations	**1860**	1216	1042	653	893	800	1418	1018	470	267	224	115
	Unique sites	**336**	255	247	206	247	242	330	244	164	123	122	73
	Entropy of mutations (%)	47.29	**63.41**	50.84	42.06	38.91	36.85	45.55	62.25	46.76	39.62	36.41	34.08
	Entropy of sites (%)	61.24	**71.70**	61.71	54.03	52.88	50.86	60.42	70.98	57.62	51.93	50.04	46.42
	Entropy of amino acids (%)	86.88	**92.68**	91.11	83.72	73.01	74.22	87.05	92.21	88.80	85.88	79.57	76.66
**Seq2Fitness**	% better than WT	**100**	**100**	**100**	**100**	**100**	**100**	**100**	**100**	**100**	**100**	**100**	**100**
	Best score	5.50	5.22	5.74	5.66	5.34	5.70	5.64	5.22	5.65	5.29	**6.30**	5.97
	100th best score	4.86	4.74	4.78	5.07	4.87	5.16	5.19	4.72	4.83	4.93	**5.89**	5.51
	1000th best score	4.54	4.13	4.46	4.66	4.59	4.69	4.88	4.03	4.49	4.60	**5.37**	5.20
	Unique mutations	1817	**5066**	1408	1096	851	1037	655	3608	840	496	449	393
	Unique sites	320	**418**	283	267	246	278	228	410	264	189	210	181
	Entropy of mutations (%)	46.35	67.69	50.48	44.02	37.88	37.36	40.54	**70.79**	47.76	41.44	37.91	35.43
	Entropy of sites (%)	60.62	71.83	61.89	57.61	53.04	52.62	54.56	**75.89**	61.78	56.88	51.54	51.68
	Entropy of amino acids	84.00	88.19	82.22	76.62	82.21	78.45	75.89	**93.23**	83.35	78.42	68.93	65.40
**Smoothed Seq2Fitness (w/GGS)**	% better than WT	57.38	75.57	**79.23**	37.21	44.66	6.63	49.00	72.97	63.04	41.76	39.19	33.12
	Best score	4.41	**5.07**	4.74	3.95	3.95	2.95	4.70	**5.07**	4.71	4.40	3.57	4.39
	100th best score	2.87	**4.38**	3.18	2.52	2.17	1.38	2.43	4.15	2.52	2.13	2.33	2.95
	1000th best score	1.62	**1.97**	1.60	1.37	1.63	0.70	1.35	1.52	1.44	1.30	1.45	1.43
	Unique mutations	3971	2937	3500	4695	5168	**5993**	3402	2735	3247	3819	3701	3955
	Unique sites	406	381	391	411	422	**424**	398	373	385	398	404	414
	Entropy of mutations (%)	62.34	**75.22**	65.73	67.37	58.69	69.63	62.42	74.95	67.86	64.79	58.22	55.53
	Entropy of sites (%)	69.87	75.90	68.57	73.77	69.47	**78.40**	71.57	75.31	72.24	72.03	69.82	68.60
	Entropy of amino acids (%)	85.19	96.03	85.33	83.84	71.44	89.24	84.96	**96.31**	88.55	83.00	75.60	80.91

Table J in S1 Text. Fuller EvoProtGrad results on alpha amylase tasks (modified code).

Fitness	Metric	**k = 1-2**	**1-3**	**1-4**	**1-5**	**1-6**
**ESM2 T = 1.0**	% better than WT	90.10	92.57	95.23	95.42	**97.00**
	Best score	18.57	25.22	33.93	34.89	**45.60**
	100th best score	13.58	17.40	21.19	24.81	**28.43**
	1000th best score	6.83	9.87	12.43	14.63	**17.27**
	Unique mutations	1743	1746	1751	**1756**	1752
	Unique sites	**346**	345	343	345	345
	Entropy of mutations (%)	**79.06**	78.11	77.67	77.62	77.47
	Entropy of sites (%)	**86.50**	85.66	85.27	85.33	85.29
	Entropy of amino acids (%)	**96.24**	96.19	96.10	96.06	96.01
**Seq2Fitness T = 1.0**	% better than WT	**7.33**	5.91	4.44	3.85	3.52
	Best score	4.84	**5.31**	4.84	4.84	3.18
	100th best score	**0.96**	0.94	0.90	0.91	0.90
	1000th best score	**0.57**	0.50	0.44	0.39	0.34
	Unique mutations	3167	3308	3405	3521	**3629**
	Unique sites	423	423	**424**	**424**	**424**
	Entropy of mutations (%)	88.51	88.60	88.57	88.87	**89.07**
	Entropy of sites (%)	98.27	98.44	98.38	98.67	**98.75**
	Entropy of amino acids (%)	99.73	99.74	99.80	99.78	**99.82**
**Smoothed Seq2Fitness (w/GGS) T = 1.0**	% better than WT	**8.32**	6.98	5.42	4.93	3.68
	Best score	4.84	**5.31**	4.84	3.18	3.18
	100th best score	0.97	**1.00**	0.96	0.95	0.86
	1000th best score	**0.52**	0.49	0.43	0.38	0.31
	Unique mutations	2744	2953	3169	3323	**3480**
	Unique sites	423	**424**	**424**	**424**	**424**
	Entropy of mutations (%)	87.01	87.36	87.83	88.30	**88.61**
	Entropy of sites (%)	98.20	98.24	98.44	98.64	**98.56**
	Entropy of amino acids (%)	99.75	99.80	**99.86**	**99.86**	99.83
**ESM2 T = 0.1**	% better than WT	97.67	98.85	99.23	99.45	**99.52**
	Best score	18.80	29.09	38.82	47.93	**54.04**
	100th best score	-	-	17.97	28.95	**38.11**
	1000th best score	-	-	-	-	-
	Unique mutations	18	19	22	**28**	25
	Unique sites	13	13	16	**20**	18
	Entropy of mutations (%)	**28.90**	28.46	27.89	28.02	27.86
	Entropy of sites (%)	29.39	32.25	33.57	34.14	**35.40**
	Entropy of amino acids (%)	76.54	76.72	75.72	75.60	**76.77**
**Seq2Fitness T = 0.1**	% better than WT	36.32	42.40	40.20	42.31	**42.60**
	Best score	4.84	4.84	4.84	3.97	**5.31**
	100th best score	0.91	1.01	1.14	1.21	**1.31**
	1000th best score	-	-	-	-	-
	Unique mutations	509	509	599	664	**713**
	Unique sites	209	206	227	231	**238**
	Entropy of mutations (%)	68.39	67.94	69.43	70.41	**71.04**
	Entropy of sites (%)	83.56	83.57	84.31	84.83	**85.53**
	Entropy of amino acids (%)	96.60	95.80	95.76	95.72	**96.81**
**Smoothed Seq2Fitness (w/GGS) T = 0.1**	% better than WT	41.49	**46.93**	44.22	42.23	44.40
	Best score	**4.84**	**4.84**	**4.84**	**4.84**	**4.84**
	100th best score	0.92	0.95	1.09	1.11	**1.23**
	1000th best score	-	-	-	-	-
	Unique mutations	330	313	414	364	**421**
	Unique sites	138	139	184	169	**187**
	Entropy of mutations (%)	63.48	62.33	65.11	63.37	**65.18**
	Entropy of sites (%)	75.35	75.64	78.18	77.76	**79.75**
	Entropy of amino acids (%)	97.84	97.39	98.17	97.96	**98.54**

Table K in S1 Text. Fuller BADASS results on NucB tasks.

Fitness	Metric	**Gamma = 0**						**Gamma = 1**					
		**k = 2-6**	**2**	**3**	**4**	**5**	**6**	**2-6**	**2**	**3**	**4**	**5**	**6**
**ESM2**	% better than WT	**100**	**100**	**100**	**100**	**100**	**100**	**100**	**100**	**100**	**100**	**100**	**100**
	Best score	45.04	16.05	23.10	30.12	38.35	**45.60**	45.00	16.05	23.10	30.97	37.74	45.10
	100th best score	40.80	13.23	21.06	28.50	35.23	41.21	41.32	13.23	21.20	28.61	35.32	**41.99**
	1000th best score	33.57	11.37	19.47	26.80	33.39	38.75	33.60	11.38	19.63	26.94	33.61	**39.96**
	Unique mutations	**1368**	791	620	528	636	685	1200	736	414	354	265	208
	Unique sites	**132**	100	93	90	96	103	128	98	80	80	75	67
	Entropy of mutations (%)	57.03	**70.43**	56.21	48.32	45.76	42.82	57.43	69.93	54.14	47.09	43.11	40.22
	Entropy of sites (%)	70.73	**74.22**	65.29	62.40	59.52	60.25	72.17	73.83	61.90	58.08	56.61	57.13
	Entropy of amino acids (%)	81.18	**90.43**	80.53	74.26	74.10	73.30	82.39	89.76	80.43	75.83	70.10	71.07
**Seq2Fitness**	% better than WT	**100**	**100**	**100**	**100**	**100**	**100**	**100**	**100**	**100**	**100**	**100**	**100**
	Best score	5.61	3.25	4.32	4.95	5.07	5.88	**6.12**	3.25	4.32	4.94	5.58	5.81
	100th best score	5.01	2.42	3.49	4.36	4.62	5.18	**5.49**	2.42	3.53	4.45	5.06	5.44
	1000th best score	4.39	1.86	3.09	3.93	4.26	4.69	4.82	1.87	3.17	4.09	4.71	**5.12**
	Unique mutations	527	**777**	469	294	289	312	380	686	264	172	165	135
	Unique sites	89	**100**	76	70	64	70	77	99	64	47	54	48
	Entropy of mutations (%)	44.93	**70.33**	54.82	46.60	43.41	41.54	45.46	69.87	52.88	46.49	43.83	41.87
	Entropy of sites (%)	57.75	**73.29**	60.46	56.61	56.75	57.44	58.19	72.65	56.87	53.43	53.94	55.40
	Entropy of amino acids (%)	76.82	**93.33**	86.86	82.32	75.73	74.27	81.45	93.20	83.22	76.53	78.49	77.14
**Smoothed Seq2Fitness (w/GGS)**	% better than WT	**100**	**100**	**100**	**100**	**100**	**100**	**100**	**100**	**100**	**100**	**100**	**100**
	Best score	5.64	3.25	4.32	4.64	5.24	5.80	5.88	3.25	4.32	4.95	5.60	**6.24**
	100th best score	5.05	2.40	3.47	4.24	4.82	5.31	5.45	2.42	3.52	4.43	5.10	**5.62**
	1000th best score	4.46	1.83	3.06	3.87	4.43	4.92	4.81	1.86	3.13	4.06	4.70	**5.26**
	Unique mutations	389	**697**	426	300	251	249	349	642	285	215	207	197
	Unique sites	81	**98**	77	67	61	63	75	96	62	55	57	55
	Entropy of mutations (%)	45.84	**70.02**	54.57	47.41	43.65	41.34	47.11	69.71	53.90	47.79	47.04	46.25
	Entropy of sites (%)	58.59	**72.70**	59.95	55.37	55.50	57.04	59.00	72.00	56.24	52.10	52.84	55.59
	Entropy of amino acids (%)	81.70	93.45	88.20	81.67	82.14	80.45	79.14	**93.48**	86.81	81.96	82.80	82.54

Table L in S1 Text. Fuller EvoProtGrad results on NucB tasks.

Fitness	Metric	**k = 1-2**	**1-3**	**1-4**	**1-5**	**1-6**
**ESM2 T = 1.0**	% better than WT	91.25	94.42	95.45	96.44	**97.04**
	Best score	14.46	20.64	23.31	27.53	**32.08**
	100th best score	10.66	14.15	17.51	20.71	**24.22**
	1000th best score	6.38	8.85	11.14	13.44	**15.90**
	Unique mutations	1256	1260	1262	1277	**1282**
	Unique sites	135	135	135	**136**	**136**
	Entropy of mutations (%)	**84.17**	82.98	82.65	82.58	82.52
	Entropy of sites (%)	**85.37**	84.00	84.02	83.89	84.19
	Entropy of amino acids (%)	**97.92**	97.62	97.44	97.20	97.03
**Seq2Fitness T = 1.0**	% better than WT	**18.24**	16.16	15.87	15.17	12.13
	Best score	2.11	2.38	2.40	2.90	**3.01**
	100th best score	0.29	0.49	0.43	**0.52**	**0.52**
	1000th best score	**-1.17**	-1.21	-1.19	-1.31	-1.47
	Unique mutations	1620	1667	1713	1767	**1770**
	Unique sites	**141**	**141**	**141**	**141**	**141**
	Entropy of mutations (%)	91.74	91.59	91.54	**91.93**	91.72
	Entropy of sites (%)	96.45	96.68	96.41	96.60	**96.79**
	Entropy of amino acids (%)	99.41	99.40	99.37	**99.55**	99.39
**Smoothed Seq2Fitness (w/GGS) T = 1.0**	% better than WT	59.47	63.45	68.14	68.67	**72.05**
	Best score	2.54	2.64	3.25	3.25	**3.97**
	100th best score	0.63	1.08	1.23	1.38	**1.52**
	1000th best score	-	-1.04	-0.89	-0.78	**-0.68**
	Unique mutations	539	587	615	622	**650**
	Unique sites	88	91	90	**94**	**94**
	Entropy of mutations (%)	77.42	77.88	78.10	78.17	**78.42**
	Entropy of sites (%)	82.70	83.07	83.33	83.37	**83.51**
	Entropy of amino acids (%)	**97.54**	97.44	97.51	97.47	97.31
**ESM2 T = 0.1**	% better than WT	92.86	96.88	98.41	98.94	**99.08**
	Best score	11.53	18.07	25.87	32.70	**40.46**
	100th best score	-	-	-	-	**10.14**
	1000th best score	-	-	-	-	-
	Unique mutations	11	17	20	**23**	22
	Unique sites	5	6	7	**9**	**9**
	Entropy of mutations (%)	25.49	28.13	30.54	31.42	**32.05**
	Entropy of sites (%)	24.32	27.81	32.91	35.72	**37.86**
	Entropy of amino acids (%)	52.77	57.50	59.38	61.48	**61.57**
**Seq2Fitness T = 0.1**	% better than WT	78.74	85.99	87.88	89.77	**91.87**
	Best score	2.69	3.58	3.96	4.01	**4.04**
	100th best score	0.85	1.40	1.75	2.24	**2.36**
	1000th best score	-	-	-	-	-
	Unique mutations	404	426	447	444	**466**
	Unique sites	83	84	85	87	**90**
	Entropy of mutations (%)	73.65	73.36	73.12	73.09	**73.74**
	Entropy of sites (%)	80.21	79.81	80.10	80.79	**81.20**
	Entropy of amino acids (%)	95.37	95.01	95.28	**95.82**	95.39
**Smoothed Seq2Fitness (w/GGS) T = 0.1**	% better than WT	85.93	92.09	92.88	**94.46**	94.18
	Best score	2.76	3.36	3.66	4.29	**4.62**
	100th best score	0.65	1.48	1.76	2.51	**2.73**
	1000th best score	-	-	-	-	-
	Unique mutations	166	172	166	**240**	231
	Unique sites	56	59	62	65	**68**
	Entropy of mutations (%)	60.63	58.52	57.70	**61.66**	61.65
	Entropy of sites (%)	71.71	69.96	70.77	72.88	**73.42**
	Entropy of amino acids (%)	**87.85**	84.21	85.06	85.54	87.36

Table M in S1 Text. Comparison of BADASS with a simpler cooling and heating regime on alpha-amylase tasks, while still updating mutation energies in the sampler

		Single cooling		Cool then heat		BADASS	
**Fitness**	**Metric**	**k=2-6**	**k=6**	**2-6**	**6**	**2-6**	**6**
**ESM2**	% better than WT	**100**	**100**	**100**	**100**	**100**	**100**
	Best score	54.09	54.12	54.10	54.54	56.50	**57.48**
	100th best score	50.50	51.97	51.44	53.42	52.00	**55.20**
	1000th best score	42.00	49.50	47.13	50.66	46.73	**52.91**
	Unique mutations	**2661**	100	1199	94	1377	120
	Unique sites	**390**	72	295	64	318	79
	Entropy of mutations (%)	**58.95**	34.24	41.25	34.45	42.37	34.54
	Entropy of sites (%)	**71.59**	48.86	55.53	47.37	57.44	46.38
	Entropy of amino acids (%)	**88.32**	76.94	79.38	77.88	87.56	84.20
**Seq2Fitness**	Best score	5.94	5.53	5.65	5.91	5.85	**6.00**
	Percent better than WT	**100**	**100**	**100**	**100**	**100**	**100**
	100th best score	5.42	5.02	5.35	5.22	5.30	**5.56**
	1000th best score	**4.99**	4.56	4.93	4.86	4.88	4.90
	Unique mutations	**578**	289	562	308	554	326
	Unique sites	**237**	144	208	149	209	161
	Entropy of mutations (%)	37.46	34.93	**38.79**	35.58	38.63	35.78
	Entropy of sites (%)	53.64	51.21	**54.95**	51.47	54.83	48.95
	Entropy of amino acids (%)	68.97	69.94	70.23	68.22	**77.90**	68.78

The simpler cool-then-heat approach cools until the sampler is unable to find enough sequences for the batch, heats until a user-specified high temperature is reached, and then repeats. The BADASS controls find the highest scoring sequence for ESM2 and Seq2Fitness on the amylase task: 57.48 versus 54.54, and 6.00 versus 5.91, respectively. But the simpler temperature control is competitive, and requires a single user-specified set point (the high temperature) rather than two, so may be preferred in some cases. We also compare BADASS to having a single cooling schedule: BADASS finds better sequences. When the mutation energies are not updated, the resulting optimization gets stuck on sequences with mediocre scores, as seen clearly in Fig A in S1 Text.

Table N in S1 Text. Comparison of EvoProtGrad results obtained using the original EvoProtGrad code and the modified code for designed sequences with only six mutations (k=6). We found that the original code led to inconsistent scores between the optimizer and rescored sequences by the fitness model. We modified the code to ensure consistency.

Fitness	Metric	Modified		Original		Original Rescored	
		T=0.1	1.0	0.1	1.0	0.1	1.0
**ESM2 Amylase**	% better than WT	99.52	97.00	100	100	98.63	98.55
	Best score	54.04	45.60	56.71	44.62	56.71	44.61
	100th best score	38.11	28.43	47.41	37.72	46.26	36.35
	1000th best score	-	17.27	-	29.56	-	28.02
	Unique mutations	25	1752	69	3374	69	3374
	Unique sites	18	345	52	405	52	405
	Entropy of mutations (%)	27.86	77.47	29.96	78.51	29.96	78.51
	Entropy of sites (%)	35.40	85.29	38.72	82.64	38.72	82.64
	Entropy of amino acids (%)	76.77	96.01	78.23	95.42	78.23	95.42
**Seq2Fitness Amylase**	% better than WT	42.60	3.52	3.05	2.49	1.56	1.57
	Best score	5.31	3.18	4.84	4.84	4.84	4.84
	100th best score	1.31	0.90	0.99	0.97	0.87	0.86
	1000th best score	-	0.34	0.63	0.59	0.45	0.45
	Unique mutations	713	3629	7181	7202	7181	7202
	Unique sites	238	424	424	425	424	425
	Entropy of mutations (%)	71.04	89.07	96.39	96.45	96.39	96.45
	Entropy of sites (%)	85.53	98.75	99.38	99.50	99.38	99.50
	Entropy of amino acids (%)	96.81	99.82	99.92	99.96	99.92	99.96
**ESM2 NucB**	% better than WT	99.08	97.04	100	100	100	98.51
	Best score	40.46	32.08	40.84	35.18	40.83	35.18
	100th best score	10.14	24.22	37.39	29.12	32.43	28.35
	1000th best score	-	15.90	-	23.99	-	22.90
	Unique mutations	22	1282	42	1677	42	1677
	Unique sites	9	136	20	141	20	141
	Entropy of mutations (%)	32.05	82.52	33.13	81.27	33.13	81.27
	Entropy of sites (%)	37.86	84.19	41.31	80.80	41.31	80.80
	Entropy of amino acids (%)	61.57	97.03	59.21	95.77	59.21	95.77
**Seq2Fitness NucB**	% better than WT	91.87	12.13	6.94	5.76	3.48	2.97
	Best score	4.04	3.01	3.66	2.33	3.66	2.33
	100th best score	2.36	0.52	0.74	0.43	0.23	0.05
	1000th best score	-	-1.47	-0.83	-0.93	-1.34	-1.44
	Unique mutations	466	1770	2675	2673	2675	2673
	Unique sites	90	141	141	141	141	141
	Entropy of mutations (%)	73.74	91.72	97.82	98.37	97.82	98.37
	Entropy of sites (%)	81.20	96.79	99.00	99.29	99.00	99.29
	Entropy of amino acids (%)	95.39	99.39	99.41	99.82	99.41	99.82

S1 Text. Appendix C: BADASS algorithm.

Algorithm A in S1 Text. Main Optimization Algorithm

Input: Budget B, Initial Temperature T_0, Reference Sequence x_{ref}, Mutation Set M
Output: Set of high-scoring sequences X_{final}
Initialize $S_{m,0} \leftarrow \{f(x_m)\}$ for all $m \in M$ ▷ Score all single mutants
Initialize $T \leftarrow T_0$
Define high and low score thresholds μ_{high}, μ_{low}
Initialize state to `Initial Transient`
for each iteration t until budget B is exhausted **do**
    Sample N sequences X_t with k mutations from $q_t(x)$
    Score sequences in X_t, update $S_{m,t}$ for all $m \in M$
    Compute μ_t and σ_t^2 from scores in X_t
    Set optimization state based on μ_t and $\mu_{\text{high}}, \mu_{\text{low}}$ ▷ See Algorithm B in S1 Text
    Update temperature T based on current state ▷ See Algorithm C in S1 Text
    Update mutation probabilities q_m using new scores and temperature
end for
Post-process: Rank all sampled sequences, select X_{final}
Return X_{final}

Algorithm B in S1 Text. State Logic for the Optimization Algorithm

function SetOptimizerState(μ_t, μ_{high}, μ_{low})
    if $\mu_t > \mu_{\text{high}}$ for last few iterations **then**
        Set state to `Active Phase Transition`
        if patience exceeded **then**
            Set state to `Phase Transition Reversal`
        end if
    else if $\mu_t < \mu_{\text{low}}$ **then**
        Set state to `Cooling Phase`
    else
        Maintain current state
    end if
end function

Algorithm C in S1 Text. Temperature Update Logic

function UpdateTemperature(T, α, α_{heat}, α_{cool}, state)
    if state is `Initial Transient` or `Active Phase Transition` **then**
        $T \leftarrow \alpha \cdot T$
    else if state is `Phase Transition Reversal` **then**
        $T \leftarrow \alpha_{\text{heat}} \cdot T$
    else if state is `Cooling Phase` **then**
        $T \leftarrow \alpha_{\text{cool}} \cdot T$
    end if
    Return T
end function

S1 Text. Appendix D: More on the temperature dependent behavior of the key quantities.

Figs 3 and 4 show how the mean sequence score and variance change versus temperature. We are also interested in a metric called n-effective and denoted by n_{eff} that describes how concentrated the sampling distribution is. It is defined as

$$n_{\text{eff}} = 2 \sum_{i=1}^{N} p_i \cdot i - 1,$$

where p_i is a probability mass function with N entries sorted from most to least likely. I.e., n_{eff} is a scaled average of the rank of the order statistic. We compute three versions: one based on the (joint: sites and amino acid) distribution over mutations, another based on the marginal of the mutation distribution over sites, and the third one based on the analogous marginal over amino acids. Figs B and C in S1 Text show these statistics versus temperature for the same datasets used in Figs 3 and 4. Changes in n_{eff} as BADASS over iterations are often leading indicators of progress, e.g., decreasing as cooling starts many iterations before the average score improves and variance changes.

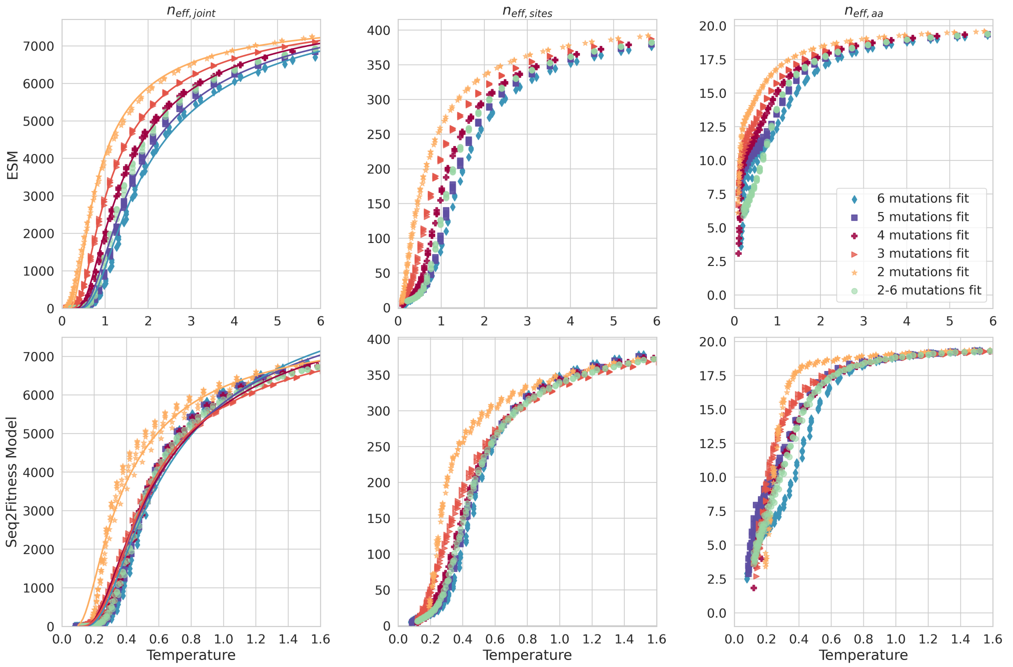

Fig B in S1 Text. The effective number of levels for the sampling distribution versus temperature for the Alpha Amylase tasks. We also show n_{eff} for the marginals over sites and amino acids, and the fit to our equations for the n_{eff} version computed from the distribution over mutations (i.e., the joint distribution of sites and amino acids).

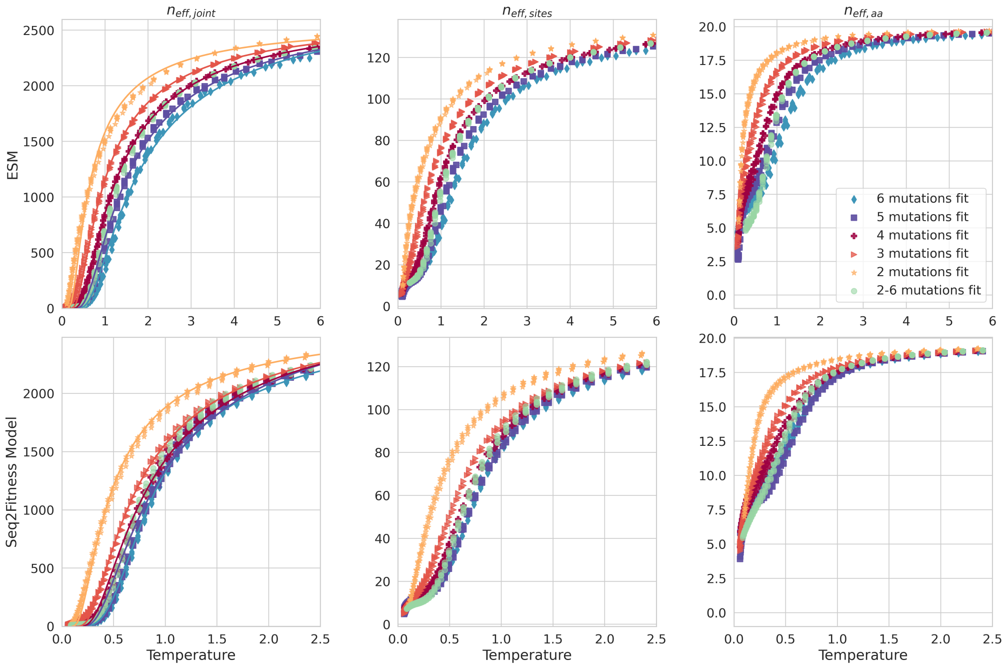

Fig C in S1 Text. The effective number of levels for the sampling distribution for the NucB tasks.

Gaussian Score Model. To understand these behaviors, we consider a simple model where N discrete energy levels E_i (negative sequence scores) are sampled i.i.d. from a Gaussian with mean μ and variance σ^2. We then define the Boltzmann distribution $\rho(E_i) = \frac{1}{Z}e^{-\beta E_i}$ that we use to sample sequences where $\beta = 1/T$ is the inverse temperature. So N is the size of the sample space we consider, e.g., $N = M$ for the distribution over mutations. We want to understand the mean, variance and n_{eff} for $\rho(E_i)$. Since the energy levels are Gaussian, we have the following expected value $\mathbb{E}[e^{-\beta E}] = e^{-\beta\mu + \frac{\beta^2\sigma^2}{2}}$. Since N is large, we use the mean field approximation

$$Z = \sum_{i=1}^{N} e^{-\beta E_i} \approx N e^{-\beta\mu + \frac{\beta^2\sigma^2}{2}}.$$

Because of the Boltzmann form of $\rho(E_i)$, the expected value $\mathbb{E}[E^m e^{-\beta E}]$ for any positive integer m can be found via:

$$\mathbb{E}[E^m e^{-\beta E}] = (-1)^m \frac{d^m}{d\beta^m}\mathbb{E}[e^{-\beta E}] = (-1)^m \frac{d^m}{d\beta^m}\left(e^{-\beta\mu + \frac{\beta^2\sigma^2}{2}}\right),$$

so $\mathbb{E}[Ee^{-\beta E}] = (\mu - \sigma^2\beta)\mathbb{E}[e^{-\beta E}]$, and $\mathbb{E}[E^2 e^{-\beta E}] = (\mu^2 - 2\sigma^2\mu\beta + \sigma^4\beta^2 + \sigma^2)\mathbb{E}[e^{-\beta E}]$. The average energy becomes

$$\langle E \rangle = \frac{\sum_{i=1}^{N} E_i e^{-\beta E_i}}{Z} \approx \frac{N(\mu - \sigma^2\beta)\mathbb{E}[e^{-\beta E}]}{N\mathbb{E}[e^{-\beta E}]} = \mu - \sigma^2\beta. \tag{1}$$

Next, we focus on the variance. A similar mean field approximation of the second moment is $\langle E^2 \rangle \approx \mu^2 - 2\sigma^2\mu\beta + \sigma^4\beta^2 + \sigma^2$, so $\text{Var}(E) \approx \sigma^2$. These mean-field results for the average energy and its variance are clearly invalid for large enough β, where we know that our distribution will concentrate most of its probability mass in the smallest energy, and have a variance of zero. So we seek a more careful approach when β is large next.

For large β, the partition function Z is dominated by the smallest energies. We use order statistics to approximate the smallest energy levels for a Gaussian distribution. For N samples from a Gaussian distribution $\mathcal{N}(\mu, \sigma^2)$, the i-th order statistic $E_{(i)}$ can be approximated by:

$$E_{(i)} \approx \mu + \sigma \Phi^{-1}\left(\frac{i}{N+1}\right) \tag{2}$$

where Φ^{-1} is the inverse cumulative distribution function (CDF) of the standard normal distribution. The smallest two energy levels are:

$$E_{(1)} \approx \mu - \sigma\sqrt{2\ln N}, \quad E_{(2)} \approx \mu - \sigma\left(\sqrt{2\ln N} - \frac{1}{\sqrt{2\ln N}}\right).$$

Using the Boltzmann distribution defined on these two energy levels yields after some algebra

$$\langle E \rangle \approx \frac{E_{(1)}}{1 + e^{-\beta(E_{(2)} - E_{(1)})}} + \frac{E_{(2)} e^{-\beta(E_{(2)} - E_{(1)})}}{1 + e^{-\beta(E_{(2)} - E_{(1)})}}$$

Simplifying since $\beta \gg 1$:

$$\langle E \rangle \approx \mu - \sigma\sqrt{2\ln N} + \frac{\sigma}{\sqrt{2\ln N}} e^{-\beta \frac{\sigma}{\sqrt{2\ln N}}}. \tag{3}$$

This stays finite as the system freezes, and goes to the expected minimum sample when N i.i.d. samples are drawn. Similarly, the second moment becomes

$$\langle E^2 \rangle \approx \frac{E_{(1)}^2}{1 + e^{-\beta(E_{(2)} - E_{(1)})}} + \frac{E_{(2)}^2 e^{-\beta(E_{(2)} - E_{(1)})}}{1 + e^{-\beta(E_{(2)} - E_{(1)})}}.$$

which simplifies when $\beta \gg 1$ to:

$$\langle E^2 \rangle \approx (\mu - \sigma\sqrt{2\ln N})^2 + \left(\frac{\sigma^2}{2\ln N} + 2\frac{\sigma}{\sqrt{2\ln N}}(\mu - \sigma\sqrt{2\ln N})\right) e^{-\beta \frac{\sigma}{\sqrt{2\ln N}}}.$$

Some algebra and keeping only leading terms in β yields

$$\mathrm{Var}(E) \approx \frac{\sigma^2}{2\ln N} e^{-\beta \frac{\sigma}{\sqrt{2\ln N}}}. \tag{4}$$

This goes to zero as the system freezes, as expected when only the lowest energy is sampled. To obtain a smooth transition between the mean-field theory and the large β behavior using order statistics, we use the interpolating function

$$w = \frac{e^{k(T - T_{\mathrm{ref}})}}{1 + e^{k(T - T_{\mathrm{ref}})}}.$$

Combining the mean and variance results of the mean field and the large β regimes with this choice results in the following functions of T :

$$\langle E \rangle = w(a + b/T) + (1 - w)c \tag{5}$$
$$\mathrm{Var}(E) = we + (1 - w)de^{-f/T}, \tag{6}$$

where a, b, c, d, e, f, k and T_{ref} are scalar parameters. These are the equations we fit to the ESM data in Figs 3 and 4. For the ML model task, the peak in variance at intermediate temperatures suggests that this simple model is not enough. But a slightly more general one, where the energy labels are sampled from two different Gaussians

rather than one, does work. The two modes would correspond to two modes of the sequence scores: one with highly scoring sequences, and the rest.

Bi-modal Gaussian Score Model. We now sample the N energy levels from the Gaussian mixture with two components $\epsilon\mathcal{N}(\mu_1, \sigma_1^2) + (1-\epsilon)\mathcal{N}(\mu_2, \sigma_2^2)$. Here, $\epsilon \ll 1$ is the fraction of samples from the first component with a high ML score, and $1-\epsilon$ is the fraction of samples from the second component with a poor ML score. So we assume $\mu_1 = \mu_2 - \delta$ with $\delta > 0$, since we think of energies rather than scores here. Proceeding similarly to the previous model with a single Gaussian component, the mean field approximation for the average energy becomes:

$$\langle E \rangle \approx \frac{N\left[\epsilon\langle Ee^{-\beta E}\rangle_1 + (1-\epsilon)\langle Ee^{-\beta E}\rangle_2\right]}{N\left[\epsilon\langle e^{-\beta E}\rangle_1 + (1-\epsilon)\langle e^{-\beta E}\rangle_2\right]} = \alpha(\mu_1 - \sigma_1^2\beta) + (1-\alpha)(\mu_2 - \sigma_2^2\beta),$$

where the subscripts 1 and 2 here denote averages with respect to the two Gaussian components $\mathcal{N}(\mu_1, \sigma_1^2)$ and $\mathcal{N}(\mu_2, \sigma_2^2)$, and where

$$\alpha = \frac{\epsilon e^{-\beta\mu_1 + \frac{\beta^2\sigma_1^2}{2}}}{\epsilon e^{-\beta\mu_1 + \frac{\beta^2\sigma_1^2}{2}} + (1-\epsilon)e^{-\beta\mu_2 + \frac{\beta^2\sigma_2^2}{2}}}$$

is a sigmoid that transitions between the two modes as a function of temperature. So the average energy is a weighted combination of the average energies of the two modes. To obtain the variance, we first find that the second moment is also the weighted second moment, i.e., $\langle E^2\rangle \approx \alpha\langle E^2\rangle_1 + (1-\alpha)\langle E^2\rangle_2$. What is interesting is that subtracting $\langle E\rangle^2$ to get the variance yields after some algebra:

$$\mathrm{Var}(E) = \alpha\sigma_1^2 + (1-\alpha)\sigma_2^2 + \alpha(1-\alpha)\delta^2. \tag{7}$$

So the variance is not just the weighted variance, and the final additional term explains the variance peak at intermediate temperatures. We also know that at low temperatures, the sigmoid α makes the first Gaussian component dominate, so we expect the mean and variance to converge to the same form obtained for the single Gaussian model based on the two lowest energies (of the first Gaussian component). This motivates the single change to the equations we fit to the Seq2Fitness tasks in Figs 3 and 4 of adding the term $w(1-w)g$ to the variance, where g is an additional parameter. More complex functions can be chosen by combining our results here, e.g., using the functional form of α for the weight, but the simpler forms suffice to capture the main aspects of the temperature dependent behavior.

Temperature dependence of n_{eff}. Finally, we derive the equation we fit to n_{eff} in Figs B and C in S1 Text. We start with a single Gaussian component model to sample N outcomes which are mutations from a Boltzmann distribution with energies E_m. So here E_m is the energy that defines the probability of sampling mutation m. We have that $p_i = \frac{e^{-\beta E_{(i)}}}{Z}$ and $Z = \sum_{i=1}^{N} e^{-\beta E_{(i)}}$, so substitution of Eq.2 yields:

$$n_{\text{eff}} \approx 2\sum_{i=1}^{N} \frac{e^{-\beta(\mu+\sigma\Phi^{-1}(\frac{i}{N+1}))}\cdot i}{\sum_{j=1}^{N} e^{-\beta(\mu+\sigma\Phi^{-1}(\frac{j}{N+1}))}} - 1$$

For large N, sums can be approximated by integrals. The denominator sum becomes:

$$\sum_{j=1}^{N} e^{-\beta\sigma\Phi^{-1}(\frac{j}{N+1})} \approx N\int_0^1 e^{-\beta\sigma\Phi^{-1}(u)}\,du$$

The numerator sum becomes:

$$\sum_{i=1}^{N} ie^{-\beta\sigma\Phi^{-1}(\frac{i}{N+1})} \approx N^2\int_0^1 ue^{-\beta\sigma\Phi^{-1}(u)}\,du$$

Using the change of variables $v = \Phi^{-1}(u)$ and $du = \phi(v)\,dv$, where $\phi(v)$ is the standard normal density function:

$$\int_0^1 e^{-\beta\sigma\Phi^{-1}(u)}\,du = \int_{-\infty}^{\infty} e^{-\beta\sigma v}\phi(v)\,dv = e^{\frac{\beta^2\sigma^2}{2}}$$

$$\int_0^1 ue^{-\beta\sigma\Phi^{-1}(u)}\,du = \int_{-\infty}^{\infty} \Phi(v)e^{-\beta\sigma v}\phi(v)\,dv \approx \Phi(-\beta\sigma)e^{\frac{\beta^2\sigma^2}{2}}$$

The last integral was approximated by the method of steepest descent. Substituting back into the expression for n_{eff}:

$$n_{\text{eff}} = 2Ne^{-\frac{\beta^2\sigma^2}{2}}\Phi(-\beta\sigma)e^{\frac{\beta^2\sigma^2}{2}} - 1 = 2N\Phi(-\beta\sigma) - 1.$$

So for small β, $\Phi(-\beta\sigma) \approx 0.5$ and $n_{\text{eff}} \approx N - 1$. For large β, using the asymptotic expansion $\Phi(-\beta\sigma) \approx \frac{e^{-\frac{\beta^2\sigma^2}{2}}}{\beta\sigma\sqrt{2\pi}}$ gives $n_{\text{eff}} \approx 2N\frac{e^{-\frac{\beta^2\sigma^2}{2}}}{\beta\sigma\sqrt{2\pi}} - 1 \approx N\frac{e^{-\frac{\beta^2\sigma^2}{2}}}{\beta\sigma\sqrt{\pi}} - 1$. Going through a similar process with the two Gaussian component model for the energies yields:

$$n_{\text{eff}} \approx 2N\left(\alpha\Phi(-\beta\sigma_1) + (1-\alpha)\Phi(-\beta\sigma_2)\right) - 1,$$

which is just the weighted combination of the per-component n_{eff}. These results motivate the functions of temperature:

$$n_{\text{eff}} = a\Phi(-\sigma/T), \text{ for the ESM task, and} \tag{8}$$
$$n_{\text{eff}} = wa_1\Phi(-\sigma_1/T) + (1-w)a_2\Phi(-\sigma_2/T) \tag{9}$$

for the Seq2Fitness task. So the first has two parameters, a and σ, and the second has six (the two sets of a and σ, and the two parameters k and T_{ref} for the weight w). The resulting fits to the joint distribution of mutations, which is a Boltzmann one, is shown on the left plot of Figs B and C in S1 Text. These equations also fit the middle plots well, even though the n_{eff} is computed over the site marginal distribution, which is not a Boltzmann. The equations do not fit the amino acid version of n_{eff} well.

The only remaining piece is the saddle point approximation used above for the integral $\int_{-\infty}^{\infty} \Phi(v)e^{-\beta\sigma v}\phi(v)\ dv$. Since $\phi(v) = \frac{1}{\sqrt{2\pi}}e^{-\frac{v^2}{2}}$, we define the exponent function $f(v) = -\beta\sigma v - \frac{v^2}{2}$. The saddle point v_0 is found by solving $\frac{df(v)}{dv} = -\beta\sigma - v = 0$, resulting in $v_0 = -\beta\sigma$. At the saddle point $f(v_0) = \frac{\beta^2\sigma^2}{2}$, so we approximate $f(v)$ near the saddle point as

$$f(v) \approx f(v_0) + \frac{f''(v_0)}{2}(v - v_0)^2 = \frac{\beta^2\sigma^2}{2} - \frac{(v+\beta\sigma)^2}{2}.$$

The integral then becomes

$$I(\beta) \approx \Phi(-\beta\sigma)e^{\frac{\beta^2\sigma^2}{2}}\int_{-\infty}^{\infty} e^{-\frac{(v+\beta\sigma)^2}{2}}\frac{1}{\sqrt{2\pi}}\,dv = \Phi(-\beta\sigma)e^{\frac{\beta^2\sigma^2}{2}}.$$

S1 Text. Appendix E: Details Of Sampler Derivation.

Here we derive the sampler distribution of Eq. 6 in the main text. Let $p_m = \sum_x I(m \in x)p(x) = \frac{1}{Z}\sum_{x \in \mathcal{N}_m} e^{f(x)/T}$ be the probability of sampling from $p(x)$ a sequence with mutation m, where $I(m \in x)$ is the indicator function of mutation m being in sequence x, and where $\mathcal{N}_m$ is the set of sequences with k mutations that have mutation m. Note that $\sum_m p_m = k$, so p_m is not a distribution over mutations; p_m/k is. Substituting the form of $q(x)$ into $D_{\mathrm{KL}}(p\|q)$ and some algebra yields:

$$D_{\mathrm{KL}}(p\|q) = -H(p) - \sum_m \log q_m \sum_x I(m \in x)p(x) = -H(p) - \sum_m p_m \log q_m. \quad (10)$$

The first term is the negative entropy of $p(x)$, which is independent of q_m and can be ignored in the optimization. The second term is the cross entropy. To minimize it, we define the objective where we add a Lagrangian multiplier to ensure the result adds up to 1 over mutations $\mathcal{L} = -\sum_m p_m \log q_m - \lambda\big(1 - \sum_m q_m\big)$. Taking the derivative w.r.t. q_m and setting it to zero gives $\partial_{q_m}\mathcal{L} = -\frac{1}{q_m}p_m + \lambda = 0$. Solving for q_m, normalizing its sum over mutations to one, and noting that $p_m = kq_m^*$ completes the derivation of Eq. 6 in the main text. Substituting q_m^* back in the cross entropy term, we find it is equal to $kH(q_m^*)$, i.e., k times the entropy of q_m^*. So $D_{\mathrm{KL}}(p\|q^*) = kH(q_m^*) - H(p) \geq 0$. Also, any other $q(x) \in \tilde{\mathcal{P}}_k$ will have a larger (or equal) $D_{\mathrm{KL}}(p\|q)$ than $kH(q_m^*) - H(p)$.

S1 Text. Appendix F: Convergence of Sampler as Temperature Drops.

Here we study how $D_{\mathrm{KL}}(p\|q)$ changes as the system is cooled for the two key distributions in the derivation of our sampler: $q^*(x)$, and its mean field approximation $\tilde{q}(x)$. These sampling distributions use the scores of all possible sequences, an assumption that makes our analysis here tractable but limits its applicability to our actual sampling distribution which approximates $\tilde{q}(x)$ with available samples. A convergence analysis for our actual sampler remains an open problem. Our goal is understanding how $D_{\mathrm{KL}}(p\|q)$ changes as the inverse temperature $\beta = 1/T$ increases. For any distribution $q(x) \in \tilde{\mathcal{P}}_k$, $D_{\mathrm{KL}}(p\|q)$ breaks up into the two terms in Eq. 10. We derive the derivative with respect to β for the two terms separately. Starting with the entropy term, we substitute $p(x)$ from Eq. 5 in the main text to find

$$H(p) = -\beta\langle f\rangle_p + \log Z,$$

where $\langle g\rangle_p$ denotes the average of any function $g(x)$ over the distribution $p(x)$, a compact notation we will use throughout this section. Simple algebra shows that the expectation of the energy is $\langle f\rangle_p = \partial_\beta \log Z$, and that the second derivative of $\log Z$ with respect to β is $\partial_\beta^2 \log Z = \sigma_f^2$, where σ_f^2 is the variance of sequence scores under $p(x)$. The latter implies $\partial_\beta\langle f\rangle_p = \sigma_f^2$. So taking the derivative of the entropy $H(p)$ with respect to β yields

$$\partial_\beta H(p) = -\beta\sigma_f^2 \leq 0. \quad (11)$$

I.e., the entropy of $p(x)$ can only decrease as the system cools, matching intuition. Next we focus on the second term in Eq. 10, which we denote here by $\ell = -\sum_m p_m \log q_m$. Substituting $p_m = kq_m^*$ into ℓ, we obtain

$$\ell = -k\sum_m q_m^* \log q_m. \quad (12)$$

So the cross entropy term in $D_{\mathrm{KL}}(p\|q)$, where the arguments are distributions over sequences, simplifies into k times the cross entropy between the optimal mutation

distribution q_m^* and the so-far arbitrary mutation distribution q_m. Next, some algebra results in

$$\partial_\beta q_m^* = q_m^* \left(\langle f|m\rangle_p - \langle f\rangle_p \right) = q_m^* \delta_m, \text{ where}$$

$$\langle f|m\rangle_p = \frac{1}{\sum_{x\in\mathcal{N}_m} e^{\beta f(x)}} \sum_{x\in\mathcal{N}_m} f(x) e^{\beta f(x)}, \text{ and } \delta_m = \langle f|m\rangle_p - \langle f\rangle_p.$$

I.e., $\langle f|m\rangle_p$ is the conditional average sequence score under $p(x)$ when restricting the sequence set to those with mutation m. Note that $\sum_m q_m^* \langle f|m\rangle_p = \langle f\rangle_p$, so $\sum_m \partial_\beta q_m^* = \langle \delta_m\rangle_{q_m^*} = 0$. Moving on, we take the derivative of ℓ:

$$\partial_\beta \ell = -k \sum_m \big(\log q_m \partial_\beta q_m^* + q_m^* \partial_\beta \log q_m \big) = -k \sum_m q_m^* \big(\delta_m \log q_m + \partial_\beta \log q_m \big). \quad (13)$$

We are ready to determine this derivative for our two sampling distributions. We start with $q^*(x) = \prod_{m\in x} q_m^*$. Since the above implies that $\partial_\beta \log q_m^* = \delta_m$, we substitute $q_m = q_m^*$ in Eq. 13 to find that

$$\partial_\beta \ell = -k \sum_m q_m^* \delta_m \log q_m^* - k \langle \delta_m \rangle_{q_m^*} = -k \mathrm{Cov}_{q_m^*} \left(\langle f|m\rangle_p, \log q_m^* \right).$$

Combining this derivative with the one for the entropy in Eq. 11 yields the expression for the change in $D_{\mathrm{KL}}(p\|q^*)$ with respect to β in Eq. 8 in the main text. Next, we work on the derivative of $D_{\mathrm{KL}}(p\|\tilde{q})$. When $\tilde{q}(x) = \prod_{m\in x} \tilde{q}_m$, we have that $\log \tilde{q}_m = \beta f_m - \log \tilde{Q}$, and $\partial_\beta \log \tilde{q}_m = f_m - \langle f_m\rangle_{\tilde{q}_m}$. Substituting these expressions in Eq. 13 and some algebra yields

$$\begin{aligned} \partial_\beta \ell &= -k\beta \sum_m q_m^* \delta_m f_m - k \log \tilde{Q} \big(\sum_m q_m^* \delta_m \big) - k \big(\langle f_m\rangle_{q_m^*} - \langle f_m\rangle_{\tilde{q}_m} \big) \\ &= -k \left[\left(\langle f_m\rangle_{q_m^*} - \langle f_m\rangle_{\tilde{q}_m} \right) + \beta \mathrm{Cov}_{q_m^*} \left(\langle f|m\rangle_p, f_m \right) \right]. \end{aligned}$$

Combining this derivative with the entropy term derivative in Eq. 11 yields the derivative of $D_{\mathrm{KL}}(p\|\tilde{q})$ with respect to β in Eq. 9 in the main text.

S1 Text. Appendix G: Low temperature approximation of convergence results.

Our goal is to approximate Eqs. 8 and 9 in the main text when $\beta \gg 1$ to leading order. Our main approximation is that sums of Boltzmann factors become concentrated on the largest one exponentially fast. Assuming there is a single sequence that attains the maximum score in $\mathcal{N}_m$ for all m to simplify, and letting $f_m^* = \arg\max_{x\in\mathcal{N}_m} f(x)$ be the largest sequence score from the set $\mathcal{N}_m$, we approximate $\sum_{x\in\mathcal{N}_m} e^{\beta f(x)} \approx e^{\beta f_m^*}$ to get

$$q_m^* \approx \frac{1}{Q^*} e^{\beta f_m^*}, \quad \text{where} \quad Q^* = \sum_m e^{\beta f_m^*}.$$

So $\log q_m^* \approx \beta f_m^* - \log Q^*$. The approximation $\sum_{x\in\mathcal{N}_m} f(x) e^{\beta f(x)} \approx f_m^* e^{\beta f_m^*}$ yields $\langle f|m\rangle_p \approx f_m^*$. So

$$\mathrm{Cov}_{q_m^*} \left(\langle f|m\rangle_p, \log q_m^* \right) \approx \beta \mathrm{Var}_{q_m^*} \left(f_m^* \right) \approx \beta \sigma_f^2,$$

The last approximation follows from the law of total variance

$$\sigma_f^2 = \mathrm{Var}_{q_m^*} \left(\langle f|m\rangle_p \right) + \langle \sigma_{f|m}^2 \rangle_{q_m^*},$$

where the last term is zero to leading order because within $\mathcal{N}_m$ the probability of sequences gets concentrated in f_m^*. Substituting this approximation in Eq. 8 in the main text we obtain Eq. 10 in the main text. Next we work on the approximation for Eq. 9 in the main text. Using the approximation for $\langle f|m\rangle_p$, we find that

$$\mathrm{Cov}_{q_m^*}\left(\langle f|m\rangle_p, f_m\right) \approx \mathrm{Cov}_{q_m^*}\left(f_m^*, f_m\right), \tag{14}$$

which is bounded in magnitude above through Cauchy-Schwartz by

$$\left|\mathrm{Cov}_{q_m^*}\left(f_m^*, f_m\right)\right| \leq \sqrt{\mathrm{Var}_{q_m^*}\left(f_m^*\right)\mathrm{Var}_{q_m^*}\left(f_m\right)} \approx \sigma_f\sqrt{\mathrm{Var}_{q_m^*}\left(f_m\right)}.$$

But we cannot express this more fully in terms of σ_f^2. Now we tackle the second term in Eq. 9 in the main text, which involves the difference of averages of f_m. We approximate q_m^* further now to only include the top two values of f_m^*, which we denote by f_1^*, and f_2^*. So

$$q_1^* \approx \frac{1}{1+e^{-\beta(f_1^*-f_2^*)}}, \quad \text{and} \quad q_2^* \approx \frac{e^{-\beta(f_1^*-f_2^*)}}{1+e^{-\beta(f_1^*-f_2^*)}}$$

for the two mutations with the two highest f_m^*, while other entries of q_m^* are zero. We similarly approximate $\tilde{q}_m$ with the two largest values of f_m:

$$\tilde{q}_1 \approx \frac{1}{1+e^{-\beta(f_1-f_2)}}, \quad \tilde{q}_2 \approx \frac{e^{-\beta(f_1-f_2)}}{1+e^{-\beta(f_1-f_2)}}.$$

Using these approximations, we compute the averages

$$\langle f_m\rangle_{q_m^*} \approx f_1^* + e^{-\beta(f_1^*-f_2^*)}(f_2^*-f_1^*), \quad \text{and} \quad \langle f_m\rangle_{\tilde{q}_m} \approx f_1 + e^{-\beta(f_1-f_2)}(f_2-f_1).$$

Taking their difference

$$\langle f_m\rangle_{q_m^*} - \langle f_m\rangle_{\tilde{q}_m} \approx (f_1^*-f_1) + e^{-\beta(f_1^*-f_2^*)}(f_2^*-f_1^*) - e^{-\beta(f_1-f_2)}(f_2-f_1),$$

which is non-negative for large enough β since $f_1^* - f_2^* \geq 0$. In the large β limit, the difference is dominated by the term $f_1^* - f_1$, with exponentially small corrections due to the second-largest values f_2^* and f_2. This shows the second term in Eq. 9 in the main text is smaller than zero in this limit. Substituting our results for both terms in the equation, we obtain Eq. 11 in the main text.

S1 Text. Appendix H: High temperature approximation of convergence results.

Here we analyze the behavior of Eqs. 8 and 9 in the main text at high temperatures, with $\beta \ll 1$. We approximate the necessary quantities to first order in β, mostly relying on the first-order Taylor approximation $e^{\beta f(x)} \approx 1 + \beta f(x)$. Applying this to q_m^*, its numerator becomes $\sum_{x\in\mathcal{N}_m} e^{\beta f(x)} \approx |\mathcal{N}_m|\left(1+\beta f_m\right)$. Similarly, the partition function is $Z \approx |S_k|\left(1+\beta\bar{f}\right)$, where $\bar{f}$ is the uniform average score over all sequences in S_k. Substituting these into the expression for q_m^*, and noting that $\frac{|\mathcal{N}_m|}{k|S_k|} = \frac{1}{M}$, where M is the total number of mutations, we get

$$q_m^* \approx \frac{1}{M}\cdot\frac{1+\beta f_m}{1+\beta\bar{f}} \approx \frac{1}{M}\left(1+\beta(f_m-\bar{f})\right).$$

Taking the logarithm of q_m^* and using the approximation $\log(1+x) \approx x$ for small x to obtain $\log q_m^* \approx -\log M + \beta(f_m - \bar{f})$. Next, we focus on the conditional expectation

$\langle f|m\rangle_p$. Substituting the first-order Taylor expansion of $e^{\beta f(x)}$ in the numerator and denominator of its definition, we have

$$\langle f|m\rangle_p \approx \frac{f_m + \beta \frac{1}{|\mathcal{N}_m|}\sum_{x\in\mathcal{N}_m} f(x)^2}{1+\beta f_m} \approx f_m + \beta \mathrm{Var}_{\mathcal{N}_m}(f(x)),$$

where the variance is based on uniform sampling of sequences in $\mathcal{N}_m$. Substituting the approximations for $\log q_m^*$ and $\langle f|m\rangle_p$ into the covariance term in Eq. 8 in the main text, we have

$$\mathrm{Cov}_{q_m^*}\left(\langle f|m\rangle_p, \log q_m^*\right) \quad \approx \quad \mathrm{Cov}_{q_m^*}\left(f_m + \beta \cdot \mathrm{Var}_{\mathcal{N}_m}(f(x)), \beta f_m\right)\right) \approx \beta \mathrm{Var}_{q_m^*}(f_m),$$

to leading order in β. Using our approximation above for q_m^*, we approximate this variance next. First, we approximate the expectations:

$$\langle f_m\rangle_{q_m^*} \approx \bar{f} + \beta \cdot \mathrm{Var}_{\mathrm{uniform}}(f_m),$$

$$\langle f_m^2\rangle_{q_m^*} \approx \langle f_m^2\rangle_{\mathrm{uniform}} + \beta \mathrm{Cov}_{\mathrm{uniform}}(f_m^2, f_m).$$

Substituting these into the variance expression and expanding to first order in β, we get

$$\mathrm{Var}_{q_m^*}(f_m) \approx \mathrm{Var}_{\mathrm{uniform}}(f_m) + \beta\big(\mathrm{Cov}_{\mathrm{uniform}}(f_m^2, f_m) - 2\bar{f}\mathrm{Var}_{\mathrm{uniform}}(f_m)\big).$$

Using the analogous small-β approximation for the Boltzmann distribution $p(x) \approx (1 + \beta(f(x) - \bar{f}))/|S_k|$, the variance of scores σ_f^2 under $p(x)$ can be expressed as:

$$\sigma_f^2 \approx \mathrm{Var}_{\mathrm{uniform}}(f(x)) + \beta \mathrm{Cov}_{\mathrm{uniform}}(f(x)^2, f(x)) - 2\beta\bar{f} \cdot \mathrm{Var}_{\mathrm{uniform}}(f(x)),$$

where $\mathrm{Var}_{\mathrm{uniform}}(f(x))$ is the variance of $f(x)$ under uniform sampling of sequences, and $\bar{f}$ is the uniform average of $f(x)$ over all sequences. So to leading order, Eq. 8 in the main text becomes

$$\partial_\beta D_{KL}(p\|q^*) \quad \approx \quad \beta\Bigg(\mathrm{Var}_{\mathrm{uniform}}(f(x)) - k\mathrm{Var}_{\mathrm{uniform}}(f_m)\Bigg). \tag{15}$$

This is small in magnitude because of β and has an ambigious sign: if $\mathrm{Var}_{\mathrm{uniform}}(f_m)$ is small then the expression is positive, e.g., if $\mathrm{Var}_{\mathrm{uniform}}(f_m) \approx \mathrm{Var}_{\mathrm{uniform}}(f(x))/|\mathcal{N}_m|$, if the scores for all sequences are approximately random. If $\mathrm{Var}_{\mathrm{uniform}}(f_m)$ is large, e.g., if the sequences of scores in the same set $\mathcal{N}_m$ are positively correlated, the expression can be negative. Next, we use the same approximations to analyze $\partial_\beta D_{KL}(p\|q^*)$ as described in Eq. 9 in the main text. Substituting the approximations, we find that

$$\mathrm{Cov}_{q_m^*}\left(\langle f|m\rangle_p, f_m\right) \approx \mathrm{Cov}_{q_m^*}\left(f_m + \beta \mathrm{Var}_{\mathcal{N}_m}(f(x)), f_m\right) \approx \mathrm{Var}_{q_m^*}(f_m) \approx \mathrm{Var}_{\mathrm{uniform}}(f_m),$$

where we drop all terms linear or higher in β, since that is all we need to recover terms to first order in Eq. 9 in the main text. The small β approximation of $\tilde{q}_m$ ends up being identical to that of q_m^* up to first order in β, so the second term in Eq. 9 in the main text is zero at this level of accuracy. Substituting the approximation above for $\mathrm{Cov}_{q_m^*}\left(\langle f|m\rangle_p, f_m\right)$ in the first term yields $\partial_\beta D_{KL}(p\|\tilde{q}) \approx \partial_\beta D_{KL}(p\|q^*)$.
