# Supplementary material for: Designing diverse and high-performance proteins with a large language model in the loop
Source: PLoS Comput Biol. 2025 Jun 5;21(6):e1013119. doi: 10.1371/journal.pcbi.1013119 (PMC12169546; doi:10.1371/journal.pcbi.1013119)
Supplement: Attachment — Submitted filename: Response to Reviewers.pdf [file pcbi.1013119.s002.pdf]

Important Note on Dataset and Resource Changes: Before addressing the specific reviewer comments, we want to note two important changes in our revised manuscript:

A. The original manuscript included analysis of the GAO protein family, which has been replaced with analysis of the NucB protein family. This change was necessary due to Solugen data usage restrictions, but it has strengthened our work by demonstrating that our methods work well with both continuous and binary fitness measurements. The NucB dataset is publicly available, making our results more reproducible.
B. The authors are no longer affiliated with Solugen Inc., where the original work was conducted. This affects our ability to perform additional computational experiments or gather new experimental data, as requested by some reviewers. We have therefore focused our revisions on maximizing the value of existing results and analyses while ensuring all claims are well-supported by the data we have. These changes do not affect the fundamental contributions or conclusions of our work, and in some ways strengthen them by demonstrating broader applicability. We have carefully addressed the reviewers' comments within these constraints, as detailed below.
C. Our code and data are already published at the indicated Github url in the paper, repeated here.

The reviewer comments are provided in black font.
Our responses are shown in blue font.
Additions to the manuscript are in red font.

Reviewer #1

1) What does "in the loop" mean in the title of the manuscript?

The phrase "in the loop" in the title refers to the iterative process of protein design implemented in our method. This process involves repeatedly querying the Seq2Fitness model during sequence sampling to evaluate fitness. Seq2Fitness integrates a protein language model at its core, enabling predictions based on both evolutionary and experimental data. The loop represents this iterative interaction where Seq2Fitness is central to guiding the optimization performed by the BADASS algorithm. We believe this encapsulates the method's innovative integration of predictive modeling and optimization.

2) When evaluating the performance of the fitness predictor, the authors compared Seq2Fitness with the very basic methods. Why not use advanced methods as baselines to compare? Such as ECNet, MODIFY and so on.

We thank the reviewer for this suggestion regarding selection of baseline methods for comparison with our method (Seq2Fitness).

Given the substantial computational resources required for model evaluations, and considering our current resource constraints since leaving Solugen Inc., we focused on comparing with carefully selected representative methods that reflect the state-of-the-art across different model categories. Specifically, we compared our approach to the augmented ESM method from Hsu et al, 2022 as a representative of semi-supervised methods, which includes several other models like ECNet. For supervised approaches, we selected the CNN method from Freschlin et al, 2024. For zero-shot methods, we selected the ESM2 zero-shot approach among others (MODIFY, MSA-transformer, EVE, or EVmutation). These selections ensure a fair and practical comparison while avoiding redundancy.

Moreover, the main aim of this work was not to conduct an extensive comparison with available methods but to emphasize the unique strengths and contributions of our approach relative to existing methods.

From our evaluations, our method showed similar performance with existing methods with a random train-test split of the data. However, we found that the key strength of our model over the baselines is the improved ability to extrapolate to new mutations and positions, which remains a core contribution of our work regardless of additional comparisons. Although including further comparisons would definitely strengthen our work, the added computational cost would be substantial, and we believe the selected methods already provide a balanced and representative evaluation of the current state-of-the-art.

We acknowledge that the rationale for the selection of the model baselines was not very clear in the manuscript, and we have modified the manuscript to make that clearer. We have also highlighted the ECNet approach and cited it in the paper.

3) The organization of the Results section seems strange. It is better to remove the sections of “The BADASS algorithm” and “Theoretical motivation for BADASS” from the Results section.

Thank you for your feedback regarding the organization of the Results section. We appreciate your suggestion to relocate the sections “The BADASS algorithm” and “Theoretical motivation for BADASS.” To improve clarity and flow, we have reframed the placement of these sections with more intuitive headings and transitions. We now combined “The BADASS algorithm” and “Theoretical motivation for BADASS” into a subsection titled: “Algorithm and Theoretical Foundation for BADASS Optimization”, signaling that these are critical to understanding the results. We also added the following transition after BADASS is described and before its motivation is explained:
“The success of BADASS is grounded in its theoretical design, which leverages principles from statistical mechanics to balance exploration and exploitation in sequence space. Here, we provide a mathematical framework that explains how BADASS sustains diversity while optimizing for high-fitness sequences. By understanding this foundation, we contextualize the algorithm’s experimental performance where its ability to avoid premature convergence and find high fitness proteins as the temperature changes becomes evident.”

We would like to note that we have received feedback from readers in the field who highlighted the importance of our mathematical expositions and the presentation of the algorithm as a key contribution of our work. We feel that it is important to include them in the Results section to ensure that readers can engage with the mathematical aspects of our methodology, which are central to our findings.

4) The manuscript lacks depth to discuss why the proposed method performs better than the state-of-the-art methods.

Thank you for your feedback regarding the depth of the discussion on why our algorithm outperforms state-of-the-art methods. In addition to previous explanations in the Introduction, Result, and Theoretical Motivation sections, we have made the following modifications to strengthen the manuscript and better explain the competitive benefits of our approach:

a. We have added a summary paragraph in the Results section that directly compares BADASS with gradient-based approaches like EvoProtGrad and GGS. This addition highlights how BADASS achieves superior performance due to its avoidance of gradient computations, dynamic sampling strategy, and maintenance of diversity, all while operating under a lower computational cost.
b. We have expanded the discussion section to highlight results from our ablation comparison that point out the advantage of our Seq2Fitness approach, particularly the use of PLM embeddings rather than one-hot or physicochemical encodings, the use of relative embeddings where we subtract the wildtype embeddings, the incorporation of zero-shot scores and the PLM logits in the

regression head, as well as the normalization of zero-shot scores. All of these unique aspects of our model contribute to enhancing its performance, relative to the models we benchmark against.

We hope these changes address your concern.

Reviewer #2

The article titled "Designing diverse and high-performance proteins with a large language model in the loop" presents and innovative approach to protein engineering. The manuscript introduces BADASS, a novel biphasic annealing algorithm, and Seq2Fitness, a semi-supervised neural network for computational protein engineering. BADASS outperforms optimization methods like MCMC-based EvoProtGrad in discovering high-fitness proteins by adjusting mutation energies and temperatures during optimization. The manuscript provides a novel strategy, and is well-written and organized.

We thank the reviewer for highlighting the novelty of our work and organization of our manuscript.

While the approach is innovative, improving the clarity of the methodology could enhance its impact. The biphasic annealing process should be clearer. The authors should consider adding a flow diagram to illustrate the optimization process better. This could also help to differentiate BADASS from MCMC methods for exploring the fitness landscape.

We appreciate the reviewer's suggestion to enhance clarity with a flow diagram. However, we believe that the algorithm is already comprehensively described in **S3 Appendix**, where we provide a step-by-step breakdown of BADASS, including initialization, sequence sampling, mutation probability updates, and temperature adjustments. Additionally, it outlines the core decision logic of the method, differentiating it from MCMC approaches. The state transition and temperature update rules are further detailed in Algorithms 2 and 3 (previously Figures 7 and 8), which we believe provide a similar function as a flow diagram, especially when combined with Fig. 1C.

Given these existing resources, adding a separate flowchart would introduce redundancy and might not provide substantial additional clarity. Instead, we propose ensuring that the relevant figures and sections are clearly referenced in the main text to guide the reader to the detailed breakdown of the methodology.

Furthermore, the authors could add details to explain why they chose a biphasic structure and dynamic mutation energies. How do these choices balance exploration and convergence of the fitness landscape?

We appreciate the reviewer's request for more details on our choice of biphasic structure and dynamic mutation energies. We have expanded the manuscript to better explain how these design choices emerge from BADASS's theoretical foundation. The algorithm approximates an intractable Boltzmann distribution using a tractable family of distributions where mutations are independent. The dynamic mutation energies and biphasic temperature control work together to optimize this approximation: high-temperature phases provide less biased samples that improve our mutation energy estimates (though at the cost of lower scores), while low-temperature phases exploit these improved estimates to find high-scoring sequences. This complementary nature is evident in our theoretical analysis and empirical results, where we observe that intermediate temperatures maximize the upper envelope of possible scores (clearly visible in Fig 2C for fixed mutation count k), suggesting an optimal trade-off between exploration and exploitation. We have added text clarifying these insights in both the Results section.

Furthermore, the authors could add more information about the evaluation metrics used in the manuscript. The Spearman correlation and adjusted AUC could be defined briefly. The authors could explain why they are well suited to the manuscript's aims.

We have included a paragraph in the Methods section with additional information about the Spearman correlation and adjusted AUC, highlighting why they are suitable for evaluating the model's performance.

The authors could also add additional information about EvoProtGrad and GGS, and what their strengths and limitations are when applied to the exploration of the fitness landscape

We appreciate the reviewer's request for additional information about EvoProtGrad and GGS. We have revised the manuscript to better explain these methods and their relationship. We now include a new paragraph in the Results section that clearly describes their shared fundamental approach using gradient-based proposal distributions, and explains both their computational limitations (requiring intensive gradient computations) and empirical performance challenges (finding fewer high-fitness sequences than BADASS). These insights previously appeared in the Supplementary Information, but we agree they are important enough to highlight in the main text. We have also maintained technical details about implementation in the SI for reproducibility. This reorganization provides readers with a clearer understanding of these methods' strengths and limitations while maintaining all relevant information.

The authors could add additional information about why BADASS achieved better scores than those algorithms, instead of performance statistics. How does the BADASS algorithm improve the “trap-prone” nature of other algorithms?

We thank the reviewer for requesting a more mechanistic explanation of BADASS's performance advantages. We have added a new paragraph in the Results section that explicitly connects BADASS's design features to its superior performance statistics. The paragraph explains how the gradient-free approach enables broader sequence space exploration, and how the biphasic temperature control targets sampling in regions that maximize the probability of finding high-fitness sequences. These mechanistic insights help readers understand why BADASS consistently outperforms alternative approaches.

The authors could include some more real-world or experimental validation of the proteins designed with their system in the manuscript to bolster its impact further.

We appreciate the reviewer's suggestion to include experimental validation of proteins designed using our system. At Solugen Inc., we implemented Seq2Fitness and BADASS to design new enzymes and validated them in the lab, achieving promising results. Over 90% of the designed proteins showed comparable or improved activity over the parent sequence, despite most mutations being absent from the training data. Unfortunately, we do not have permission to publish this data, and we no longer have access to Solugen's experimental resources. While we agree that experimental validation would greatly enhance the work, we believe the strong performance of our method on existing datasets demonstrates the value of our method, and would be a promising basis for future research.

Overall, the manuscript presents a novel approach to computational protein engineering, with advancements over existing methods. Enhancing the clarity of the methodology and providing additional context around the evaluation metrics, algorithmic choices, and performance would improve its impact. Incorporating experimental validation would make this manuscript even more robust. The work has the potential to become a valuable reference.

We thank the reviewer for pointing out that our work has the potential to be valuable reference in the

literature. As pointed above, we have addressed all the reviewer's points and made major modifications to the manuscript to provide additional context to improve the clarity.

Reviewer #3

Recap of paper

• The paper presents a new innovative approach to protein optimization by integrating semi supervised machine learning into a pLM (Seq2Fitness) as well as an annealing strategy for promoting exploration of the fitness landscape (BADASS).

Strengths

• pLM fitness optimization: The Seq2Fitness model uses embeddings from ESM2 to predict fitness landscapes. It combines evolutionary data with experimental labels, and allows extrapolation vs interpolation from training data. It tries to address the challenge of limited experimental data by utilizing the rich information encapsulated in PLMs. This is a slightly different approach than the typical ways to RL or fine tuning to align the pLM to the experimental data.

• The paper introduces a dynamic biphasic annealing strategy for efficient sequence sampling, which maintains diversity while preventing premature convergence. The balance between cooling and heating phases is well-explained theoretically - and is shown to work in the insilico optimization experiments. It promotes exploration to obtain diverse high-fitness sequences. The strategy is sound, in particular with recent papers describing very rugged fitness landscapes observed in experimental dataset (Sandhu 2024).

• While the current work focuses on enzymes, the optimization algorithm seems reusable with any pLM, and any style of fine tuning, generally useful for any sequence fitness optimization problem.

We thank the reviewer for highlighting the strengths of our work, particularly in enabling in-silico navigation of rugged protein fitness landscapes. To further reinforce this point, we have included a citation to Sandhu et al. (2024) in our manuscript.

Weaknesses – and suggestion for improvements

• The Seq2Fitness model is benchmarked against base ESM2. It is expected the semi supervised model will win – which is also shown clearly in later evaluations. A benchmark against typical techniques to adapt a pLM to experimental data would be good to get a fair comparison. Paper references (Hsu 2022, Nature) that has a list of good options, as does many other papers references. This could be classical fine tuning, LORA, or some reinforcement learning scheme like Preference Optimization. I would be very interested to seeing the difference.

We used both Seq2fitness and ESM2 as fitness predictors with our optimization algorithm (BADASS), as a demonstration of semi-supervised and unsupervised application. We note that this was not to argue that one was better than the other, but to illustrate that both supervised and self-supervised models could be used with the BADASS algorithm. To benchmark Seq2Fitness, we compared it with relevant state-of-the-art models in the literature, including the semi-supervised approach from Hsu et al (2022), and the supervised CNN method from Freschlin et al (2024), also adapting a pLM. We acknowledge that

this may not be very clear in the manuscript, and have now revised the Result and Methods sections to clarify this point.

We also acknowledge that fine-tuning the ESM model, with LoRA or other parameter-efficient methods, could potentially improve performance. However, for Seq2Fitness, we opted for using a light regression head on frozen ESM embeddings due to the huge computational requirements of fine tuning, and to enable efficient training and evaluation across multiple train-test splits. We note that in this work we were able to train our Seq2Fitness models with a 24GB GPU using a batch size of 400. In contrast, fine-tuning with LoRA even when applied to only the last 8 layers of ESM exhaust 32GB of GPU memory with batch sizes as small as 4 on proteins of similar size as ours (Sledzieski2024, 10.1073/pnas.2405840121).

We recognize from the recent literature, that fine-tuning pLMs with parameter-efficient fine-tuning methods, such as LoRA, can significantly enhance performance. These fine-tuning techniques can be seamlessly integrated into our Seq2Fitness framework and BADASS optimizer. Accordingly, we have updated the discussion to reference relevant literature and recommend that readers consider incorporating these approaches, depending on their computational budget.

• To show the semi-supervised model works, another option is to run against a standard benchmark. The ProteinGym benchmark dataset (Notin, Neurips 2023) is a very commonly used benchmark, containing a collection of 200+ DMS experiments. Getting a comparative evaluation on this set would be very helpful, and would help compare directly with many more methods (including ESM2 zero shot).

Training and evaluating across the extensive 217 datasets in ProteinGym would be computationally expensive, and currently exceeds our resource constraints as we no longer have access to the computational infrastructure at Solugen where this work was originally conducted. As a result, we decided to focus our analysis on four representative protein datasets. We specifically selected these four datasets as they are rich in multi-mutant data, addressing our objective of evaluating models capable of handling epistatic inference and predicting the fitness effects of variants with multiple mutations relative to the wild type. Whereas ProteinGym predominantly consists of single-mutants, our chosen benchmark datasets (GB1, AAV, NucB and Amy-Bacsu) are well-established in the literature and representative of real-world use cases, including enzymes, binding, and viral proteins. The addition of the NucB dataset (which replaced our original GAO protein analysis) particularly strengthens our work by demonstrating effectiveness on binary fitness measurements. We believe that these datasets provide a robust foundation for assessing the prediction capabilities of our method on fitness landscapes. While we are no longer able to run additional computational experiments due to resource constraints, we believe our analysis on these four diverse datasets provides strong evidence of our method's effectiveness.

• Experimental validation of BADASS: The paper uses 4 datasets for insilico fitness optimization as benchmarks (please described in more details e.g. in the suplementary material). Proper experimental validation invitro would make the paper much stronger. However, since not all groups have access to wet lab experimentation, it is an unfair ask. I can only suggest joining some of the optimization competitions to prove the worth of the method.

We appreciate the reviewer's suggestion for experimental validation to enhance our work. As mentioned in our response to Reviewer #2, we would be eager to conduct such experiments but currently lack access to experimental resources. Nonetheless, we believe the strong results presented in this paper, especially the superior performance of our method on existing published datasets, are compelling and scientifically valuable to be published as a stand-alone paper. At the reviewer's request, we have expanded the manuscript to include additional details about the four selected datasets.

• As an alternative, although optimizing 4 backbones help focus on the individual datasets, it does not show the types of datasets that could be opttimized (potentially also antibodies, binders, etc). Testing on more -and more diverse- experimental backbones and different types of experimental assays would be useful to prove the generality of the method - but I would only encourage and it would not be a requirement.

We agree that testing on a broader range of protein classes would help demonstrate the generality of the method. This was particularly why we selected the four fitness datasets to reflect a diversity of protein types. Amy_Bacsu and NucB are enzyme datasets, AAV is a viral protein, and GB1 is an immunoglobin binding protein. This diverse selection was an attempt to highlight the applicability of our method across distinct protein categories. We have updated the manuscript to more clearly explain this rationale.

• Limited benchmarking against alternative strategies: the method is benchmarked against EvoProdGrad and GSS. However, many other optimization approaches exist, that also attempts to optimize towards one or more virtual fitness scores. E.g Lambo2, Guided discrete diffusion (Gruver et al, 2023), or Discrete Walk Jump sampling (Frey et al, 2023). A comparison on the gain in virtual fitness against such competing methods would be interesting - or a method described in Hsu et al 2024.

We appreciate the reviewer's suggestion to benchmark BADASS against additional optimization methods. We note that our choice to compare against EvoProtGrad and GGS was deliberate, as these represent the current state-of-the-art in protein sequence optimization using language models, compared to other approaches. Both methods share a fundamental approach (gradient-based proposals) but represent different optimization strategies - EvoProtGrad's direct gradient-based sampling and GGS's attempt to smooth the fitness landscape. Their comparative performance thus provides insight into the limitations of gradient-based approaches generally.

We acknowledge the existence of other protein sequence optimization methods such as Lambo2 and guided discrete diffusion. However, we note that we no longer have access to the computational resources at Solugen where this work was conducted, and are unable to conduct additional benchmarking, which would significantly delay publication without likely changing our core findings. However, we have strengthened our evaluation in other ways, particularly by demonstrating our method's effectiveness on the newly added NucB dataset, which provides evidence of our approach's versatility across both continuous and binary fitness landscapes. We feel that our theoretical analysis and empirical results already demonstrate BADASS's key advantages - namely, its ability to efficiently explore sequence space without gradient computations while maintaining diversity through dynamic temperature control.

We have added citations in our manuscript to the alternative methods you brought to our attention to provide readers with a complete context of the field, while maintaining our focus on the most relevant comparisons that illuminate BADASS's innovations: “Several approaches have been proposed to address this challenge, including samplers based on diffusion models [13, 14], and gradient-based methods like EvoProtGrad and GGS [15, 16]. However, common optimization techniques often struggle to efficiently navigate the vast sequence space [17, 18].”

Minor corrections

• Metrics: I believe the tables with metrics can be condensed significantly. The Top 100 metric and % Better than WT are not needed (perhaps just a line with the WT fitness on a figure?).

We appreciate the reviewer’s suggestion to condense the tables and their efforts to improve clarity. However, we respectfully disagree with the removal of the *100th best score* metric and the overall condensation of the tables in the main text.

The *100th best score* provides critical insight into the optimization process by balancing between the absolute best-performing sequences (*best score*) and broader sequence diversity (*1000th best score*). Removing this metric would obscure a key aspect of our method's performance, particularly when comparing sequence fitness distributions across different approaches. Unlike *best score*, which may reflect an outlier, or *1000th best score*, which captures broad trends, the *100th best score* provides a meaningful snapshot of the optimization's reliability in consistently identifying high-fitness sequences.

We acknowledge that some of the detailed metrics tables in the Appendix could be streamlined. As requested by the editor, we will move them to a separate SI document. However, the tables in the main text are already curated to present key performance metrics concisely, and condensing them further risks omitting essential comparative information.

While adding a line for WT fitness in a figure could provide a useful reference point, it does not replace the need for explicit reporting of *% Better than WT* in tables. The latter provides a quantitative measure of performance across an entire sequence set, which is more informative than a single visual marker in a plot. We appreciate the reviewer's input and hope this explanation clarifies our rationale.

Additional, typical criteria used of designability and e.g. structural metrics are not included - to show mutations do not reduce structural integrity (scored with Alphafold pLDDT).

As both AlphaFold and ESM2 rely on sequence-based evolutionary and co-evolutionary data, mutations with very high ESM2 scores (which are utilized by the BADASS optimizer) are very likely to correspond to high pLDDT scores as well, potentially making the use of pLDDT somewhat redundant. We felt that structural metrics that incorporate force-field or thermodynamic-based evaluations, such as FoldX or Rosetta, may provide better assessment of structural fidelity. However, due to their significant computational costs, we did not evaluate these methods in our study. We appreciate the reviewer's suggestion and have expanded the discussion section to highlight this as a potential consideration for the reader seeking to apply our method.
