# Supplementary material for: Designing diverse and high-performance proteins with a large language model in the loop
Source: PLoS Comput Biol. 2025 Jun 5;21(6):e1013119. doi: 10.1371/journal.pcbi.1013119 (PMC12169546; doi:10.1371/journal.pcbi.1013119)
Supplement: Attachment — Submitted filename: Response to Reviewers V2.pdf [file pcbi.1013119.s003.pdf]

Response to reviewers' comments

The reviewer comments are provided in black font.
Our responses are shown in blue font, with manuscript additions in italics and between quotation marks.

Reviewer #1
My comments were addressed.

Reviewer #2
The authors have sufficiently responded to the comments in their revision. They provide a novel protein engineering approach to design proteins with machine learning, integrating a semi-supervised neural network prediction model and an optimization algorithm to create novel amino acid sequence

Reviewer #4
The authors present Seq2Fitness, a supervised deep learning model that integrates per-residue embeddings and log-probabilities from the ESM2 language model with a dual-path convolutional architecture and an MLP to predict protein variant fitness. The proposed method performs well on selected benchmarks and is further integrated into the authors' sequence design framework, BADASS. The overall idea is solid, and the combination of zero-shot signals with learned features is effective. However, the following concerns should be addressed before the manuscript is considered for acceptance:

We thank the reviewer for their positive view of our work and for highlighting that the method is effective. We address all raised concerns below.

• The authors state: “For example, for both protein families 100% of the top 10,000 sequences found by BADASS have higher Seq2Fitness predictions than the wildtype sequence, versus less than 11% for competing approaches.” Both BADASS and Seq2Fitness are methods developed by the authors and are tightly integrated for directed evolution. Since BADASS directly depends on Seq2Fitness for scoring, comparing its output to sequences generated by competing approaches—especially when evaluation is based on Seq2Fitness predictions—is potentially biased and not a fair comparison. This limitation should be acknowledged.

At the reviewer’s suggestion, we have modified the manuscript to better point out the independence of our optimization and fitness prediction methods. We note that BADASS is an independent optimization algorithm that operates as a stochastic sampler over model predictions and is not inherently tied to any specific fitness predictor. In our work, we demonstrated the effectiveness of BADASS with both the semi-supervised model we proposed (Seq2Fitness) as well as with zero-shot predictions of the ESM model, showing that BADASS can work with any fitness predictor. Researchers can easily apply BADASS to other fitness models by modifying a few lines of code. Alternatively, they may choose to use our Seq2Fitness

model with a different optimization algorithm. Neither the fitness predictor nor the optimization method is coupled to the other.

Accordingly, now the Evaluating BADASS section includes the following: "*All optimization methods in our comparison (BADASS, EvoProtGrad, and GGS) were evaluated using the same fitness functions for both optimization and evaluation, which is standard practice in directed evolution optimization. BADASS demonstrates superior performance regardless of whether the fitness function is Seq2Fitness or ESM2, indicating that its effectiveness is not tied to any particular scoring model but rather stems from its efficient exploration of sequence space.*"

• The model is trained and evaluated on datasets corresponding to few proteins. As a result, Seq2Fitness is not currently generalizable across proteins. This important limitation should be clearly stated wherever the performance or applicability of the algorithm is described.
• It would be helpful if the authors could comment on the potential of training Seq2Fitness on larger and more diverse datasets, such as ProTherm, which contains mutation data across a wide range of human proteins, including both single and multiple mutations. This could significantly improve the model's generalizability.

At the reviewer's suggestion, we've updated the manuscript to more clearly state that our current results are limited to the datasets used and that broader generalization would require further evaluation on additional protein such as ProTherm and ProteinGym (duly cited). Now the Discussion includes: *"It is important to note that the current implementation of Seq2Fitness requires protein-specific training for each reference sequence of interest. Unlike zero-shot models such as ESM2, which can be applied to any protein sequence without additional training, Seq2Fitness models are trained specifically for each protein family being studied, which enables higher prediction accuracy at the cost of reduced generalizability."* We also added ProTherm as a reference: *"To enable further advances in*
protein design, the field should invest in generating and sharing annotated, multi-mutant
datasets across diverse protein families and functions [39, 40]."

However, we would like to highlight that we evaluated Seq2Fitness and BADASS on four datasets representing a range of protein types: viral proteins, enzymes, and binding proteins (AAV, GB1, AMY-BACSU, and NucB). The consistent performance across these diverse proteins suggests that the methods are applicable to different protein classes.

We agree that expanding the evaluation to more datasets would be valuable. However, this would come with substantial computational cost. In fact, many notable studies in the field report results on fewer datasets. For instance, Ding et al. evaluated their method on a single dataset (GB1), and Kirjner et al. used two (AAV and GB1). Compared to these, our work provides one of the broader and more diverse evaluations currently available.

• The manuscript includes the following sentence: "ESM2 Model: a zero-shot model for scoring protein sequences based on their unsupervised representations. We use the ESM2 650M

model, and the mutant marginal score as the fitness. (add equation here?)”
Remove the phrase “(add equation here?)”

We thank the reviewer for catching this. We have removed the phrase from the manuscript.

• The authors note: “We evaluated designed sequences containing 2–6 mutations relative to a reference sequence.”
Is the reference sequence periodically updated? If not, it would be useful to explain why the reference sequence was not iteratively updated during sequence generation. Iterative refinement of the reference is a standard approach in directed evolution and could improve sequence exploration.

We thank the reviewer for this suggestion. In our work, the reference sequence is fixed and corresponds to the wildtype or starting sequence. Unlike evolutionary algorithms that iteratively update the reference by accumulating mutations over time, our optimization approach samples all k-mutation variants in a single pass. The process doesn't involve stepwise mutation accumulation. Instead, only the number of generated sequences increases from iteration to iteration (based on batch size), not through iterative refinement of a single reference. We have modified the manuscript to better point this out. Specifically, we added to the Algorithm and Theoretical Foundation for BADASS Optimization section: *"Unlike evolutionary algorithms that iteratively update the reference by accumulating mutations over generations, BADASS maintains a fixed reference sequence (typically the wildtype) throughout the optimization process. This approach enables efficient exploration of the k-mutation neighborhood around a single reference point, rather than stepwise mutation accumulation. The optimization process expands the number of sampled sequences with each iteration while maintaining the same reference point."*

• Finally, the evaluation of Seq2Fitness is based on an 80/20 train-test split. It would be more appropriate to use 5-fold cross-validation to obtain a more robust estimate of model performance, especially given the relatively small dataset sizes. While leave-two-out cross-validation may be computationally expensive, 5-fold cross-validation is a reasonable and standard compromise.

We appreciate the reviewer’s suggestion regarding cross-validation. While 5-fold cross-validation would provide more robust performance estimates, we chose 80/20 train-test splits given the computational demands of training these models on large datasets, which would require roughly five times the compute. Moreover, we no longer have access to computational resources at Solugen, where this work was done, making additional experiments impractical. Consistent with the literature, we expect the relative performance of the models to remain stable between 80/20 splits and k-fold cross-validation. To better capture real-world performance and assess generalization more thoroughly, we evaluated the models using mutational, positional, and distance-based splits. These more challenging evaluation scenarios provide more stringent

tests of model performance than standard cross-validation and better represent the real-world use case of designing novel protein sequences.

We have modified the manuscript to note the tradeoff between compute limits and stronger evaluation of 80/20 vs. k-fold validation, and to highlight our focus on testing real-world generalization. Under Predicting Protein Fitness with Seq2Fitness, we added: *"We used 80/20 train-test splits for computational efficiency, although 5-fold cross-validation could provide more robust performance estimates with sufficient computational resources. Our focus on challenging extrapolation tests (mutational, positional, and two-vs-rest splits) provides stringent evaluation of real-world generalization capability, which is the primary concern in protein engineering applications."*
